# Applications of Artificial Intelligence in Oral and Maxillofacial Cosmetic Surgery: A Systematic Review of Diagnostic, Planning, and Outcome Assessment Tools

**DOI:** 10.7759/cureus.92185

**Published:** 2025-09-12

**Authors:** Hussain M Abdali, Ahmed Y Ayoub, Rahaf A Omer, Raghad A Alanazi, Baraah S Alsaedi, Lama B Almutairi, Bayan A Alhakami, Abdulrahman Y Alfifa, Nasser A Alnasser, Alaa H Hakami

**Affiliations:** 1 College of Medicine, Jazan University, Jazan, SAU; 2 College of Medicine, Northern Border University, Arar, SAU; 3 College of Medicine, Taibah University, Madinah, SAU; 4 College of Medicine, Qassim University, Buraydah, SAU; 5 College of Medicine, Alrayan Colleges, Madinah, SAU; 6 College of Medicine, King Saud University, Riyadh, SAU

**Keywords:** artificial intelligence, cosmetic surgery, diagnostic support, machine learning, neural networks, oral and maxillofacial surgery, orthognathic surgery, outcome assessment, surgical planning, third molar extraction

## Abstract

Artificial intelligence (AI) has rapidly expanded into oral and maxillofacial cosmetic surgery, offering potential improvements in diagnosis, surgical planning, perioperative risk assessment, and outcome prediction. Despite promising results, the extent of its clinical utility and methodological rigor across published studies remains unclear. This systematic review synthesized and critically appraised the applications of AI in oral and maxillofacial cosmetic surgery, focusing on diagnostic support, preoperative planning, and esthetic or functional outcome evaluation. A comprehensive search of PubMed, Cochrane, Scopus, and Web of Science from inception to November 2024 identified 11,031 records, of which 14 studies were included after screening. Data extraction captured study characteristics, AI techniques, input modalities, comparators, and outcomes, and the study quality was appraised using the PROBAST-AI tool. The included studies encompassed diverse AI applications. In third molar surgery, convolutional neural networks achieved 78.9-90.2% accuracy for extraction difficulty, while neural networks predicted postoperative swelling with 98% accuracy. In orthognathic diagnostics, models using cephalograms and facial photographs reported accuracies above 90%, with specificity up to 99%. For surgical planning, AI predicted soft-tissue changes with sub-millimeter error margins, outperforming conventional models. Perioperative risk models predicted blood loss with mean errors <10 mL, while aesthetic applications quantified age reduction post-rhinoplasty and generated simulations with high surgeon agreement. Despite these advances, most studies were retrospective, single-center, and limited by small or homogeneous datasets, with an overall high risk of bias. Overall, AI demonstrates strong potential for enhancing diagnostic accuracy, surgical planning, risk prediction, and esthetic evaluation in oral and maxillofacial cosmetic surgery. However, current evidence is constrained by methodological weaknesses, limited validation, and ethical concerns, including dataset bias and subjective outcome measures. Future research should prioritize prospective multicenter validation, standardized reporting frameworks, multimodal data integration, and transparent model design to enable safe and effective clinical translation.

## Introduction and background

Artificial intelligence (AI), particularly machine learning (ML) and deep learning (DL), has ushered in a paradigm shift in healthcare by enabling advanced image analysis, predictive modeling, and decision support across various disciplines [1]. In medicine, AI approaches - especially convolutional neural networks (CNNs) - often outperform traditional methods in image-based diagnostics and outcome prediction. Dentistry and oral surgery are no exception, as AI gains ground in image interpretation, automated segmentation, and simulation workflows [2,3].

In oral and maxillofacial surgery (OMFS), AI is increasingly applied to improve diagnostics, surgical planning, and prognostic assessment. Innovations include computer-assisted radiographic analysis, more accurate risk prediction, and streamlined postoperative management. The drive toward AI automation reflects both the demand for precision and the challenges posed by complex anatomy and the need for aesthetically pleasing outcomes [4].

One of the most promising areas is orthognathic surgery, where accurate simulation of post-treatment facial morphology is critical for planning and patient counseling [5]. DL models using geometric morphometrics, 3D facial scans, and cephalometric data have demonstrated sub-millimeter prediction errors, underscoring AI’s potential to enhance surgical precision and visual communication [6,7].

AI is also advancing the automation of labor-intensive image-processing tasks. DL-based platforms for automatic segmentation of maxillofacial CT images markedly reduce clinician workload by accelerating preoperative processing and aligning dental with anatomical models. Such automation decreases human error while optimizing planning time and resources [8,9].

Despite these advances, major challenges hinder routine clinical use. Most studies rely on retrospective, single-center datasets, lack external validation, and face class imbalance, particularly in rare or severe cases. These limitations compromise reproducibility, generalizability, and clinical credibility, highlighting the need for standardized reporting (e.g., TRIPOD+AI, CONSORT-AI) and multicenter validation [2,3].

Given this evolving landscape, a systematic evaluation of AI applications in oral and maxillofacial cosmetic surgery is both timely and necessary. This review synthesizes current evidence on AI-based models for diagnostic support, preoperative planning, and outcome prediction in facial aesthetic and corrective procedures, with the aim of guiding future research toward clinically robust, validated AI tools in OMFS.

## Review

Methods

Literature Search Strategy

This review followed the Preferred Reporting Items for Systematic Reviews and Meta-Analyses (PRISMA) guidelines [10]. A comprehensive search was conducted in PubMed, Cochrane Central Register of Controlled Trials (CENTRAL), Scopus, and Web of Science (WOS), covering all records from inception to July 5, 2025. Search terms combined controlled vocabulary and free-text keywords: (“Artificial Intelligence” OR “Machine Learning” OR “Deep Learning” OR “Neural Network*” OR “Convolutional Neural Network*” OR “Generative Adversarial Network*” OR “AI” OR “ML” OR “DL”) AND (“Oral Surgery” OR “Maxillofacial Surgery” OR “Orthognathic Surgery” OR “Orthodontics” OR “Rhinoplasty” OR “Reconstructive Surgical Procedures” OR “Dentofacial Deformities” OR “Third Molar Extraction” OR “Nasal Reconstruction”) AND (predict* OR diagnos* OR classif* OR assess* OR plan* OR simul* OR forecast* OR outcome* OR risk). Filters restricted results to English-language human studies.

Eligibility Criteria

Selection criteria were defined using the PICOS (population, intervention, comparison, outcome, study design) framework [9]. Eligible studies were English full-text original articles that involved patients undergoing oral and maxillofacial cosmetic or corrective procedures, applied AI methods for diagnosis, surgical planning, or outcome prediction, used conventional approaches, expert assessments, or statistical models as comparators where available, and reported quantitative outcomes related to diagnostic accuracy, predictive performance, or clinical utility. Excluded were reviews, case reports, conference abstracts without full text, non-human studies, and articles published in languages other than English.

Study Selection

Two reviewers independently screened titles and abstracts according to the eligibility criteria. Full texts of potentially relevant articles were then assessed in detail. Discrepancies were resolved through discussion, with a third reviewer consulted when necessary.

Data Extraction

For each included study, the following information was extracted: author and year of publication, country, study design, participant characteristics, sample size, AI approach, input data (e.g., imaging, clinical factors), primary outcomes, and main findings. Extraction was performed independently by two reviewers, with disagreements resolved through discussion or third-party adjudication.

Quality Appraisal

Methodological quality and risk of bias were independently assessed by two reviewers using the PROBAST-AI tool, an adaptation of the original Prediction model Risk Of Bias ASsessment Tool (PROBAST) framework for AI prediction model studies [11]. The tool evaluates four domains, namely, participants, predictors, outcomes, and analysis, rating each as “low,” “high,” or “unclear” risk of bias, followed by an overall judgment. AI-specific considerations - such as dataset representativeness, validation methods, and overfitting - were also incorporated. Disagreements were resolved by consensus.

Results

Study Selection

**Figure 1:**
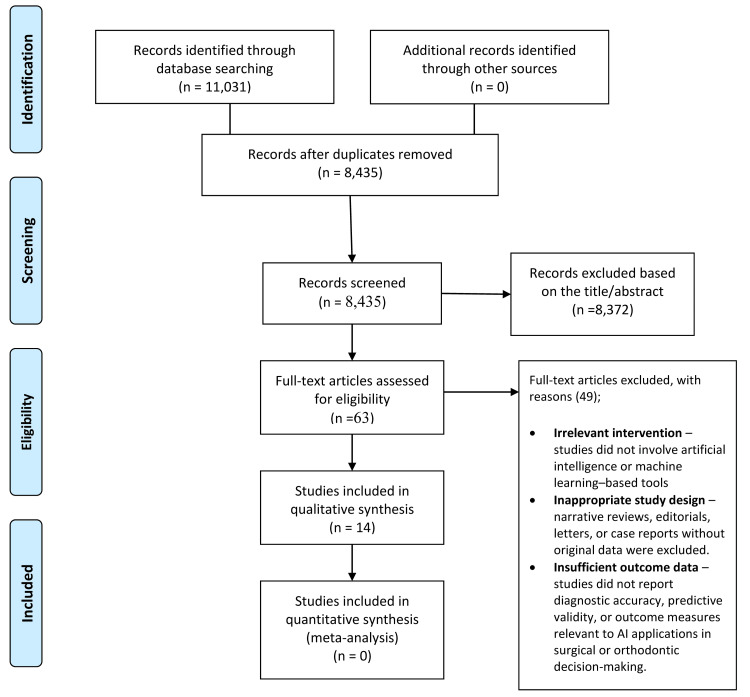
PRISMA 2020 flow diagram for the study selection in the systematic review on artificial intelligence in oral and maxillofacial cosmetic surgery PRISMA: Preferred Reporting Items for Systematic Reviews and Meta-Analyses

Database searches identified 11,031 records (PubMed, n = 2,470; Cochrane, n = 538; Scopus, n = 3,456; Web of Science, n = 4,567), with no additional records from other sources. After duplicate removal, 8,435 unique records remained. Screening excluded 8,372 as irrelevant, leaving 63 full-text articles for assessment. Of these, 49 were excluded for lacking an AI/ML component, unsuitable design (e.g., reviews, case reports), or insufficient outcome data. Fourteen studies met the inclusion criteria and were included in the qualitative synthesis; none were eligible for quantitative meta-analysis (Figure 1).

Study Characteristics

The 14 included studies spanned multiple regions, including China, Japan, Korea, Argentina, the USA, the Netherlands, and Austria (Table 1). Designs ranged from small prospective feasibility studies (e.g., Zeng et al. [5]) to large retrospective cohorts (e.g., Kim et al. [12]; Stehrer et al. [13]). Sample sizes varied widely, from five patients with nasal defects [5] to datasets exceeding 22,000 images [6].

**Table 1:** Summary of the included studies on the applications of artificial intelligence in oral and maxillofacial cosmetic surgery Abbreviations: AI = Artificial Intelligence; ML = Machine Learning; DL = Deep Learning; CNN = Convolutional Neural Network; GAN = Generative Adversarial Network; OMF = Oral and Maxillofacial; 3D = Three-Dimensional; CT = Computed Tomography; CBCT = Cone-Beam Computed Tomography; MRI = Magnetic Resonance Imaging; ROC = Receiver Operating Characteristic; AUC = Area Under the Curve

Study ID (Author, Year)	Country/Setting	Study Design	Population/Sample	Intervention (AI Tool)	Comparator/Reference	Outcomes Measured	Main Results
Choi et al., 2019 [1]	Republic of Korea (Seoul National University & Korea University)	Retrospective diagnostic ML study	316 orthodontic patients (160 surgical, 156 non-surgical); exclusion of asymmetry, deformities, prior treatment	ANN model (2-layer ANN, cephalogram measurements + indices) for surgery vs. non-surgery and extraction decisions	Specialist orthodontist diagnosis (gold standard, >10 yrs experience)	Accuracy of surgery vs. non-surgery, type of surgery, extraction vs. non-extraction	Surgery vs. non-surgery: 96%; Surgery type: 100%; Extraction decision: Class II = 97%, Class III = 88%; Final success rate: 91%
Shin et al., 2021 [2]	Korea (Wonkwang University, Daejeon Dental Hospital)	Retrospective AI model development + validation	840 patients with dentofacial dysmorphosis/malocclusion (mean age: 23.2; 461 M/379 F)	CNN (ResNet34 backbone, PA + lateral cephalograms) for predicting necessity of orthognathic surgery	Specialist consensus (2 orthodontists, 3 surgeons, 1 radiologist)	Accuracy, sensitivity, specificity	Accuracy 0.954; Sensitivity 0.844; Specificity 0.993; inference time ~64 ms
Ter Horst et al., 2021 [3]	Netherlands (Radboud Univ. Medical Centre)	Retrospective model development & validation	133 mandibular hypoplasia patients treated with BSSO advancement (119 training, 14 test; mean age: 29.5 yrs; 53% female)	DL autoencoder neural network using 3D photographs + CBCT mandibular displacement data	Mass Tensor Model (MTM) biomechanical simulation (IPS CaseDesigner®)	MAE, RMSE, % simulations ≤1 mm and ≤2 mm error across lower face, lip, chin	DL outperformed MTM: MAE lower face 1.0 mm vs 1.5 mm (p=0.022); ≤1 mm accuracy: 64.3% vs 21.4%; chin MAE 1.4 mm vs 2.0 mm; clinically acceptable (<2 mm)
Zhang et al., 2018 [4]	China (Wuhan Univ. School & Hospital of Stomatology)	Retrospective model development & validation	400 patients undergoing impacted mandibular third molar extraction (300 train, 100 test)	ANN (conjugate gradient BP) trained on 15 personal, anatomical, surgical factors	Clinical swelling assessment (baseline vs. 72 h post-op)	Prediction accuracy, classification of swelling (mild, moderate, severe)	ANN achieved 98% accuracy in predicting swelling; test set: 98/100 correctly predicted; cross-validation + early stopping improved generalization
Zeng et al., 2018 [5]	China (West China Hospital of Stomatology, Sichuan University)	Brief clinical prospective feasibility study	5 male patients with total nasal defects, ages 32–43 (mean: 36.4)	Database search + 3D image registration + dimensionality reduction algorithm for virtual planning of forehead flap reconstruction	Empirical forehead flap planning (traditional surgeon’s experience)	3D model accuracy (difference pre- vs post-op), subjective similarity ratings by 10 evaluators	Mean absolute error 0.92–1.04 mm; max error 3.12–4.07 mm; majority of evaluators rated results as “similar/relatively similar”
Borsting et al., 2020 [6]	USA (UC Irvine; Case Western Reserve; University Hospitals Cleveland Medical Center)	Development + validation study with mobile deployment	Training: 22,686 pre-/post-op rhinoplasty photos (public source); test: 2,269 unseen images; 8 clinicians rated 2,053 images	“RhinoNet” CNN (MobileNetV2, transfer learning, deployed as mobile app) to classify rhinoplasty status (before vs. after)	Expert consensus ratings (plastic surgery attendings and residents)	Accuracy, sensitivity, specificity, Cohen’s Kappa, diagnostic likelihood ratios	Accuracy 85%; Sensitivity 0.84, Specificity 0.83; comparable to experts (Sens. 0.81, Spec. 0.87); Kappa ~0.67 (model) vs. 0.68 (experts); best-case AI+expert concordance 0.87
Yoo et al., 2021 [7]	Korea (Wonkwang Univ. Dental Hospital)	Retrospective diagnostic AI study	600 panoramic radiographs (1053 mandibular third molars; mean age: 27.5 ± 9.1 yrs; 305 M/295 F)	CNN (ResNet-34 pretrained on ImageNet) for ROI detection & difficulty classification (Pederson Difficulty Score)	Consensus PDS from 3 experts (oral surgeon, resident, radiologist)	Accuracy, sensitivity, specificity, Cohen’s kappa, RMSE	Accuracy: 78.9% (C1), 82.0% (C2), 90.2% (C3); Kappa: 70.9%, 65.2%, 85.5%; RMSE = 0.67; performed best for mid-range scores, overestimated low difficulty, underestimated very high difficulty
Lee et al., 2020 [8]	Korea (Korea University Ansan Hospital)	Retrospective diagnostic model validation	333 patients (159 orthodontic treatment, 174 orthognathic surgery; mean age: 23.1 ± 5.1; 181 F/152 M)	DCNNs (Modified-Alexnet, MobileNet, ResNet50) on cephalometric radiographs; Grad-CAM for explainability	Clinician-based cephalometric evaluation	Accuracy, sensitivity, specificity, AUC of models; visualization of regions of interest	Modified-Alexnet: Accuracy 91.9% (AUC 0.969, Sens. 0.852, Spec. 0.973); MobileNet: Acc. 83.8% (AUC 0.908); ResNet50: Acc. 83.8% (AUC 0.923). Grad-CAM focused on maxillary/mandibular teeth, mandible, and symphysis
Kim et al., 2021 [12]	Korea (Seoul National University Dental Hospital)	Retrospective diagnostic study with 5-fold cross-validation	960 patients (640 orthodontic only; 320 orthognathic surgery); age 15–37 yrs (mean: 24.6)	CNNs (ResNet-18, -34, -50, -101) applied to cephalometric radiographs (landmarks auto-detected with WebCeph)	Expert orthodontic diagnosis (gold standard)	Accuracy, sensitivity, specificity, AUC (ROC curves)	ResNet-18: Accuracy 93.8%, AUC 0.979; ResNet-34: Accuracy 93.6%, AUC 0.974; ResNet-50: Accuracy 91.1%, AUC 0.945; ResNet-101: Accuracy 91.3%, AUC 0.944. Shallower networks (18, 34) outperformed deeper ones
Stehrer et al., 2019 [13]	Austria (Kepler Univ. Linz), with Zurich & Straubing collaboration	Retrospective cohort with ML model development/validation	950 patients (2006–2017); excluded SARPE, <18 yrs, bleeding disorders, RBC transfusions	Random Forest predicting perioperative blood loss (demographic, hematologic, surgical variables)	Actual perioperative blood loss (hemoglobin balance method)	Correlation between predicted vs. actual blood loss; feature importance	Strong correlation (p < 0.001). Mean difference: 7.4 ml (SD 172.3). Key predictors: bimaxillary surgery, pre-op hematocrit/hemoglobin, surgical time, blood volume
Chinski et al., 2022 [14]	Argentina (Otolaryngology Center & University of Buenos Aires)	Cross-sectional survey study	97 otolaryngologists (residents and specialists) evaluated 1,436 image simulations	GAN-based deep learning model trained on 1,200 surgeon-generated rhinoplasty simulations	Surgeon-generated image simulations (traditional manual editing)	Agreement with simulated images (7-point Likert scale); correlation and Bland-Altman analysis	Median agreement score: Surgeon = 6 (IQR 5–7), AI = 5 (IQR 4–6), p < 0.0001; evaluators in total/partial agreement 68.4% (AI) vs. 77.3% (surgeon); Spearman correlation 0.92
Dorfman et al., 2020 [15]	USA (UCLA, Division of Plastic and Reconstructive Surgery)	Retrospective chart review with ML analysis	100 female patients, 16–72 yrs (mean 32.8); post-op follow-up ≥12 weeks (mean: 29 weeks)	ML (ranking CNN via Microsoft Azure Face API) to estimate apparent age from pre- and post-op photos	Actual chronological age and pre-op vs. post-op patient images	Age estimation accuracy, correlation with real age, anti-aging effect of rhinoplasty	CNN predicted pre-op age within 0.03 yrs of actual (r = 0.91); patients appeared on avg 3.1 yrs younger post-op (p < 0.0001)
Jeong et al., 2020 [16]	Korea (Wonkwang University, Daejeon Dental Hospital)	Retrospective AI model development + validation	822 patients with dentofacial dysmorphosis/malocclusion (mean age: 23.5; 452 M/370 F)	CNN (VGG19 backbone, global pooling, facial photos) for predicting necessity of orthognathic surgery	Specialist consensus (2 orthodontists, 3 surgeons, 1 radiologist) based on cephalometry	Accuracy, precision, recall, F1 score	Accuracy 0.893; Precision 0.912; Recall 0.867; F1 = 0.889; visualization maps highlighted lips, teeth, chin
Tanikawa et al., 2021 [17]	Japan (Osaka Univ. Dental Hospital & Kanomi Dental Hospital)	Retrospective study with model development & 11-fold cross-validation	137 patients: Surgery group (n=72, mean age 23.5 yrs, Le Fort I + BSSO) and Orthodontic group (n=65; mean age: 15.6 yrs; 4 premolar extractions)	Two AI systems: System S (orthognathic) & System E (orthodontic) combining GMMs + deep learning on cephalograms and 3D stereophotogrammetry	Actual pre- and post-treatment 3D morphology	System error (mm), success rate at <1 mm and <2 mm, error distribution	Mean error: 0.89 mm (S), 0.69 mm (E). Success rate <1 mm: 54% (S), 98% (E); <2 mm: 100% both. Errors greatest at nasal alar, chin, mouth corner (S), and lower lip (E). Both clinically acceptable (<2 mm)

Most studies applied DL or neural network-based models tailored to specific contexts. In orthognathic and orthodontic applications, CNNs and autoencoder-based methods analyzed cephalograms, CBCT scans, and 3D photographs [2,3]. In oral surgery, Yoo et al. [7] classified third molar extraction difficulty with a CNN, while Zhang et al. [4] used an artificial neural network to predict postoperative swelling. Aesthetic and reconstructive applications included GAN-based rhinoplasty simulations [14], CNN-driven apparent age estimation [15], and automated pre-/post-rhinoplasty classification [6].

Comparators were typically expert clinician assessments or conventional planning methods. For example, Choi et al. [1] and Kim et al. [12] benchmarked diagnostic models against orthodontists’ decisions, while Jeong et al. [16] validated CNN classifications against consensus specialist diagnoses. In reconstructive contexts, Zeng et al. [5] compared AI-assisted forehead flap planning with traditional empirical approaches. Similarly, Chinski et al. [14] tested AI-generated rhinoplasty simulations against surgeon-generated ones, while Borsting et al. [6] and Dorfman et al. [15] compared AI outputs to expert consensus or chronological age. These studies underscore AI’s role as an adjunct rather than a replacement for clinical decision-making.

Outcomes emphasized technical performance metrics such as accuracy, sensitivity, specificity, area under the curve (AUC), and error margins. Many models achieved >90% accuracy in classification tasks [2,12]. Aesthetic and reconstructive studies reported millimeter-level error margins [3,5] or agreement scores with expert raters [14]. Some explored novel endpoints, such as quantifying age reduction post-rhinoplasty [15] or predicting perioperative blood loss [13]. Overall, outcomes highlight AI’s versatility but also its reliance on surrogate or short-term measures rather than validated patient-reported or long-term clinical endpoints.

Quality Assessment

Most studies had notable methodological limitations. Small or narrowly defined cohorts were common (e.g., Zeng et al. [5]; Ter Horst et al. [3]), while larger datasets (e.g., Shin et al. [2]; Tanikawa et al. [17]) were often restricted to single-center, ethnically homogenous populations. Even Stehrer et al. [13] and Borsting et al. [6], despite large samples, were limited by single-institution settings or selection bias (Table 2).

**Table 2:** Risk of bias assessment of the included studies using the PROBAST-AI tool Abbreviations: AI = Artificial Intelligence; ML = Machine Learning; DL = Deep Learning; CNN = Convolutional Neural Network; ANN = Artificial Neural Network; GAN = Generative Adversarial Network; OMF = Oral and Maxillofacial; 3D = Three-Dimensional; CT = Computed Tomography; CBCT = Cone-Beam Computed Tomography; MRI = Magnetic Resonance Imaging; ROI = Region of Interest; CV = Cross-Validation; ROC = Receiver Operating Characteristic; AUC = Area Under the Curve

Author (Year)	Participants	Predictors	Outcomes	Analysis	Overall
Choi et al., 2019 [1]	Low – 316 patients (160 surgical, 156 non-surgical), Korean population	Low – 12 cephalometric measures + 6 indexes, well-defined and standardized	High – Outcomes limited to surgery/non-surgery classification, no long-term patient outcomes	High – Retrospective, single-institution, ANN model with internal split but no external validation	High
Shin et al., 2021 [2]	High – Only Korean patients from one hospital, limited representativeness	Low – Cephalograms with clear definitions and standardized preprocessing	High – Outcome based on specialist consensus, may introduce subjectivity	High – Retrospective, no external validation, single-center dataset	High
Ter Horst et al., 2021 [3]	High – Small test set (n=14), single-center, limited representativeness	Low – Input data (3D photographs, CBCT-based mandibular displacements) clearly defined	Low/Moderate – Outcome (soft tissue displacement) measured objectively with 3D photographs	High – Retrospective, very small validation set, no external validation, potential overfitting	High
Zhang et al., 2018 [4]	Low/Moderate – 400 patients (300 training, 100 testing), single Chinese hospital	Low – 15 clearly defined predictors (demographics, anatomy, surgical factors)	Low – Objective swelling measured with facial landmarks and nylon thread	High – Internal split-sample validation, 5-fold CV, no external validation	High
Zeng et al., 2018 [5]	High – Only 5 male patients, very limited sample, no diversity	Low – Imaging and database-based predictors clearly defined	High – Outcomes based on similarity ratings and error measurements, not standardized patient-reported or validated scales	High – Retrospective design, no external validation, reliance on in-house algorithms	High
Borsting et al., 2020 [6]	Low – Large dataset (22,686 images) from RealSelf, biased toward successful cases	Low – Clear imaging predictors (pre-/post-op photos, standardized preprocessing)	High – Outcome defined as rhinoplasty status, subject to bias from idealized uploads	High – Retrospective, no external clinical validation beyond expert comparison	High
Yoo et al., 2021 [7]	Moderate – 600 panoramic radiographs, 1053 molars, single Korean center	Low – ROI detection + CNN (ResNet-34), standardized panoramic features	Moderate – Extraction difficulty scored with Pederson Difficulty Score (subjective consensus)	High – Train/test split, internal validation, no external validation	High
Lee et al., 2020 [8]	Low – 333 patients (174 surgery vs. 159 orthodontic), single-center Korean cohort	Low – Cephalometric radiographs analyzed with DCNNs, standardized preprocessing	High – Outcome restricted to binary classification (surgery vs. orthodontic), no long-term validation	High – Retrospective, 4-fold CV, no external validation, risk of overfitting	High
Kim et al., 2021 [12]	Low – 960 patients (640 orthodontic vs. 320 surgical), single-center Korean cohort	Low – Cephalometric radiographs, standardized landmark-based preprocessing	High – Outcome limited to binary classification, no patient-reported outcomes	High – Retrospective, 5-fold CV + small external test set, no independent validation	High
Stehrer et al., 2019 [13]	Low – Large sample (950 patients) but single-center, retrospective, possible selection bias	Low – Demographic, surgical, and hematologic predictors clearly defined	High – Outcome (perioperative blood loss) estimated via hemoglobin balance, not direct measurement	High – Retrospective, internal validation only, no external validation	High
Chinski et al., 2022 [14]	High – Non-probabilistic sample (97 otolaryngologists at a single meeting), risk of selection bias	Low – Predictors (surgeon vs. AI simulations) clearly defined using standardized image pairs	High – Outcome based on subjective Likert agreement scores, no standardized clinical outcome	High – Cross-sectional survey, no external validation, AI trained on single-surgeon dataset	High
Dorfman et al., 2020 [15]	Low – 100 female patients, age range 16–72 (exclusion of males limits generalizability)	Low – CNN-based imaging predictors clearly specified (Microsoft Azure Face API, ranking CNN)	High – Outcomes limited to algorithm-estimated "apparent age," not standardized clinical measures	High – Retrospective, single-surgeon cohort, no external validation	High
Jeong et al., 2020 [16]	Low/Moderate – 822 patients (411 surgery vs. 411 non-surgery), single-center Korean cohort	Low – Facial photographs (frontal and lateral) standardized, preprocessing described	High – Outcome based on cephalometric skeletal criteria, no functional validation	High – Retrospective, internal train/test split, no external validation	High
Tanikawa et al., 2021 [17]	High – Small sample (n=137; 72 surgery, 65 extraction), limited to Japanese patients	Low – 3D facial semi-landmarks + cephalometric landmarks, clearly defined	Low/Moderate – Outcome was 3D post-treatment morphology measured objectively	High – 11-fold CV only, no external validation, possible overfitting	High

Predictors were generally well specified and imaging-based (e.g., standardized cephalograms [8,12] or preprocessed photographs [16]), supporting reproducibility. However, most studies used single data modalities without integrating multimodal or functional parameters, limiting clinical applicability.

Outcomes were frequently subjective or narrow. For example, Chinski et al. [14] and Dorfman et al. [15] relied on subjective ratings or algorithm-derived apparent age rather than standardized patient-reported measures. Others, such as Lee et al. [8] and Jeong et al. [16], restricted endpoints to binary classifications, with no validation against functional or quality-of-life outcomes. Nearly all studies were retrospective, using internal rather than external validation, resulting in an overall high risk of bias. These limitations highlight the need for multicenter, prospective studies with standardized clinical and patient-centered outcomes.

Effect of AI for Third Molar Surgery

AI applications in third molar surgery primarily addressed extraction difficulty and postoperative outcomes. Yoo et al. [7] developed a CNN based on the Single Shot Multibox Detector with a ResNet backbone, achieving 78.9-90.2% accuracy across Pederson Difficulty Score criteria, with best performance for angulation (90.2%) and lowest for depth (78.9%). This was the first fully automated object detection and classification model for this task, enhancing efficiency via mini-batch learning. Clinically, it supported preoperative planning and training, though it could not account for surgeon skill or clinical nuances.

Zhang et al. [4] applied an optimized backpropagation neural network to predict postoperative swelling, achieving 98% classification accuracy with faster convergence than conventional methods. By stratifying swelling severity, the model provided practical support for counseling and perioperative planning, though it lacked volumetric quantification and external validation. Together, these studies illustrate AI’s utility for both predictive (difficulty) and prognostic (swelling) tasks, albeit with limited generalizability.

Effect of AI in Orthognathic Surgery: Diagnostics and Screening

AI diagnostic models consistently achieved >90% accuracy in distinguishing surgical from orthodontic cases [1,2,8,12,16]. Shin et al. [2] demonstrated 95.4% accuracy and 99.3% specificity by fusing lateral and posteroanterior cephalograms. Kim et al. [12] found shallower ResNet-18 networks outperformed deeper models (93.8% accuracy; AUC: 0.979). Facial photograph-based models [16] reached 89% accuracy, supporting their feasibility as low-cost screening tools. Choi et al. [1] reported 96% accuracy in surgical decision-making using stepwise neural networks, though camouflage vs. surgical correction remained challenging in Class II malocclusions. Collectively, these findings position AI as a reliable diagnostic adjunct in orthognathic surgery.

Effect of AI for Surgical Planning and Soft-Tissue Prediction

AI has shown strong promise in surgical planning, particularly soft-tissue prediction. Tanikawa et al. [17] achieved sub-millimeter accuracy in predicting 3D facial changes, confirming near-linear relationships for mandibular setback (86-100%) but variability in lip adaptation (40-60%). Ter Horst et al. [3] demonstrated that deep learning reduced prediction error by 33% compared to conventional mass tensor models, achieving ~1 mm accuracy in lower facial predictions. Together, these studies highlight AI’s superiority over traditional planning, with applications in counseling, expectation management, and osteotomy refinement. Limitations include small cohorts and a lack of real-time or multimodal integration.

Effect of AI for Perioperative Risk and Safety

Stehrer et al. [13] applied random forest models to predict blood loss in orthognathic surgery, achieving a mean error of 7.4 mL in 950 cases. Key predictors included bimaxillary procedures, male sex, and operative time. Although useful for transfusion planning and perioperative efficiency, the retrospective design and absence of external validation limit clinical adoption.

Effect of AI in Aesthetic and Reconstructive Surgery

In rhinoplasty, Borsting et al. [6] reported AI classification of post-surgical patients with κ = 0.67, nearly identical to expert consensus (κ = 0.68). Dorfman et al. [15] showed that AI-based age estimation quantified a mean 3.1-year reduction in apparent age post-rhinoplasty. Chinski et al. [14] reported strong agreement (ρ = 0.92) between AI-generated and surgeon-generated simulations, though experts rated human simulations slightly higher.

In reconstructive surgery, Zeng et al. [5] provided early evidence of AI-assisted nasal defect restoration planning, achieving ~1 mm error between planned and postoperative reconstructions. Although limited by very small samples, these findings illustrate AI’s growing role in both cosmetic and reconstructive surgery, particularly for outcome evaluation and planning.

Discussion

This systematic review synthesized evidence on the applications of AI in oral and maxillofacial cosmetic surgery, focusing on third molar surgery, orthognathic diagnostics, surgical planning, perioperative risk, and aesthetic/reconstructive outcomes. Across the included studies, AI consistently demonstrated high performance in diagnostic classification, surgical planning, and outcome prediction, with accuracies often exceeding 85-95%. In third molar contexts, CNNs and other neural architectures achieved 78.9-98% accuracy in predicting extraction difficulty and postoperative swelling, aligning with prior findings [4,7]. Similarly, diagnostic studies reported accuracies above 90% for distinguishing surgical versus orthodontic cases, with specificity approaching 99% when combining cephalometric modalities [2,12]. These results underscore AI’s potential to improve decision-making, enhance patient safety, and serve as a reliable adjunct to clinical expertise.

Despite these promising results, most studies were retrospective, single-center, and limited by small sample sizes or lack of external validation, restricting generalizability. While AI shows immediate clinical relevance in diagnostic support, surgical planning, and perioperative risk estimation, its integration into real-world practice is not yet established. More prospective, multicenter studies with standardized reporting and external validation are needed to confirm reproducibility and assess clinical utility. Taken together, current evidence suggests that AI has emerged as a valuable tool for oral and maxillofacial cosmetic surgery, but its role remains that of a decision-support adjunct rather than a replacement for clinician expertise.

Limitations

Most included studies were retrospective, single-center, and based on small, homogeneous datasets, limiting external validity. The absence of standardized reporting frameworks (e.g., TRIPOD-AI, CONSORT-AI) reduced transparency and comparability. Restricting inclusion to English-language studies may have introduced publication bias, while heterogeneity in AI models, input modalities, and outcome measures precluded meta-analysis. Ethical concerns - including data bias, limited interpretability, and cultural subjectivity in aesthetic assessment - remain important barriers to clinical translation.

## Conclusions

AI is rapidly advancing in oral and maxillofacial cosmetic surgery, with growing evidence for its value in diagnostics, surgical planning, perioperative risk prediction, and esthetic outcome assessment. Reported accuracies often exceed those of conventional approaches, yet most studies remain preclinical, retrospective, and single-center, limiting generalizability. Methodological shortcomings, lack of standardized reporting, and unresolved ethical challenges - particularly bias and interpretability - further constrain clinical adoption. Future research should emphasize prospective, multicenter trials, integration of multimodal datasets, and robust ethical safeguards to ensure transparent, equitable, and safe translation of AI into routine practice.
